# Prebreeding studies of leaf rust resistant Triticum aestivum/T. timopheevii line L624

**DOI:** 10.18699/VJGB-23-73

**Published:** 2023-10

**Authors:** S.N. Sibikeev, I.G. Adonina, A.E. Druzhin, O.A. Baranova

**Affiliations:** Federal Center of Agriculture Research of the South-East Region, Saratov, Russia; Institute of Cytology and Genetics of the Siberian Branch of the Russian Academy of Sciences, Novosibirsk, Russia Kurchatov Genomic Center of ICG SB RAS, Novosibirsk, Russia; Federal Center of Agriculture Research of the South-East Region, Saratov, Russia; All-Russian Research Institute of Plant Protection, Pushkin, St. Petersburg, Russia

**Keywords:** Triticum aestivum/T. timopheevii line, 2AS.2AL-2AtL translocation, leaf rust resistance, impact on productivity and grain quality, Triticum aestivum/T. timopheevii линия, 2AS.2AL-2AtL транслокация, устойчивость к листовой ржавчине, влияние на продуктивность и качество зерна

## Abstract

Triticum timopheevii Zhuk. attracts the attention of bread wheat breeders with its high immunity to the leaf rust pathogen. However, introgressions from this species in Triticum aestivum L. are little used in practical breeding. In the presented study, the agronomic value of T. aestivum/T. timopheevii line L624 was studied in comparison with the parent cultivars Saratovskaya 68, Dobrynya and the standard cultivar Favorit during 2017–2022. Introgressions from T. timopheevii in L624 were detected by the FISH method with probes pSc119.2, pAs1 and Spelt1, as well as microsatellite markers Xgwm312, Xgpw4480 and Xksum73. Translocations of 2AS.2AL-2AtL and on 2DL were detected as well. Line L624 is highly resistant to Puccinia triticina both under the background of natural epiphytotics and under laboratory conditions. PCR analysis with the DNA marker of the LrTt1 gene (Xgwm312) revealed that it is not identical to the Lr gene(s) in L624. According to a five-year study, the grain yield of L624 was, on average, higher than that of Favorit and Dobrynya, but lower than that of Saratovskaya 68. Line L624 had a lower weight of 1000 grains than the recipients, and was at the same level with the standard cultivar Favorit. Introgressions from T. timopheevii in L624 increased the grain protein content by comparison with Saratovskaya 68 and Favorit, but it was at the same level as in Dobrynya. As for parameters of flour and bread, L624 was not inferior to the recipient cultivars, but by volume and porosity of bread, it surpassed Saratovskaya 68. Moreover, L624 surpassed Favorit by the elasticity of the dough, the ratio of the elasticity of the dough to the extensibility and the strength of the flour. Thus, the results obtained suggest that introgressions in chromosomes 2A and 2D in L624 do not impair baking properties.

## Abstract (ru)

Вид Triticum timopheevii Zhuk. привлекает внимание селекционеров мягкой пшеницы высоким иммунитетом к возбудителю листовой ржавчины. Однако интрогрессии от этого вида в T. aestivum L. мало используются в практической селекции. В представленном исследовании изучена агрономическая ценность T. aestivum/T. timopheevii линии Л624 по сравнению с родительскими сортами Саратовская 68, Добрыня и сортом-стандартом Фаворит в течение 2017–2022 гг. Интрогрессии от T. timopheevii у Л624 выявлены с помощью метода FISH с зондами pSc119.2, pAs1 и Spelt1, а также микросателлитных маркеров Xgwm312, Xgpw4480 и Xksum73. Обнаружены транслокации 2AS.2AL-2AtL и в длинном плече хромосомы 2D. Линия Л624 высокоустойчива к Puccinia triticina как на фоне естественной эпифитотии, так и в лабораторных условиях. С использованием ПЦР-анализа с ДНК-маркером гена LrTt1 (Xgwm312) установлена его неидентичность Lr-гену(ам) у линии Л624. По данным пятилетнего изучения, урожайность зерна у Л624 была в среднем выше, чем у сортов Фаворит и Добрыня, но ниже, чем у сорта Саратовская 68. По массе 1000 зерен Л624 уступала реципиентам и была одного уровня с сортом-стандартом Фаворит. Интрогрессии от T. timopheevii у Л624 увеличили содержание белка в зерне по сравнению с сортами Саратовская 68 и Фаворит, но с сортом Добрыня оно было на одном уровне. В целом по показателям качества муки и хлеба линия Л624 не уступила сортам-реципиентам, а по объему и пористости хлеба превзошла Саратовскую 68. В то же время Л624 превышала сорт-стандарт Фаворит по упругости теста, отношению упругости теста к растяжимости и силе муки. Таким образом, полученные результаты позволяют сделать предположение, что интрогрессии в хромосомах 2А и 2D у линии Л624 не ухудшают хлебопекарные свойства

## Introduction

Leaf rust (caused by the fungus Puccinia triticina Eriks.) is a harmful disease both in Russia and abroad. Despite the fact that the importance of this disease has decreased for a number of grain-growing Russian regions, the damage remains quite extensive (Gultyaeva et al., 2021). The production of resistant bread wheat cultivars makes it possible to avoid economically significant damage to plants by this pathogen. Resistance to P. triticina in bread wheat is controlled by Lr-genes. To date, 82 Lr-genes have been identified (McIntosh et al., 2022), but most of them have been overcome by the pathogen. In general, the following genes are used in Russian bread wheat cultivars: Lr1, Lr3, Lr9, Lr10, Lr19, Lr20, Lr24, Lr26, Lr34, Lr37 and Lr6Agi1, Lr6Agi2, LrSp. These genes are used in various combinations, but only the LrSp, Lr6Agi1, and Lr6Agi2 genes are not overcome (Gultyaeva et al., 2021).

The low genetic diversity of effective resistance genes to the leaf rust pathogen can be solved by involving species related to bread wheat in hybridization. Thus, out of 82 identified Lr-genes, 39 were transferred from alien species (McIntosh et al., 2013, 2018, 2022). Triticum timopheevii Zhuk. (AtAt GG, 2n = 28) is one of the sources of effective resistance genes. This species is very popular among breeders because of its unique high resistance to a complex of diseases. Both in Russia and abroad, numerous attempts have been made to transfer pathogens resistance genes from T. timopheevii into bread and durum wheat by direct hybridization followed by backcrossing to cultivated species (Allard, Shands, 1954; Jørgensen, Jensen, 1972; Skurygina, 1984; Tomar et al., 1988; Kozlovskaya et al., 1990; Budashkina, Kalinina, 2001; Brown-Guedria et al., 2003; Singh et al., 2017).

In addition, synthetic alloploid forms from crossing T. timopheevii with Aegilops tauschii Coss. were produced as an intermediate form – a “bridge”. As a result, forms with 2n = 42 and genome composition AtAtGGDD – T. kiharae Dorof. et Migusch. were obtained (Dorofeev et al., 1979), as well as the synthetic of Dr. Savov (Leonova et al., 2007). With similar hybridization with the natural mutant T. timopheevii – T. militinae Zhuk. et Migusch, a synthetic form Triticum miguschovae was obtained (Zhirov, Ternovskaya, 1984).

Subsequently, when bread wheat was crossed with these synthetics, a number of lines that were resistant to leaf and stem rust pathogens were obtained. Thus, a set of pathogenresistant lines in the gene background of the Saratovskaya 29 cultivar, the so-called С29 immune (С29im), as well as the spring bread wheat cultivar Pamyati Maistrenko, was obtained at the Institute of Cytology and Genetics of the Siberian Branch of the Russian Academy of Sciences (Laikova et al., 2013).

However, despite numerous studies, only two Lr-genes from T. timopheevii have been identified – Lr18 in the 5BS.5BL-5G#1L translocation and Lr50 localized on 2BL (McIntosh et al., 2013). In addition, the “Catalogue of Genes Symbols for Wheat” (McIntosh et al., 2013) lists three Lr-genes with a temporary (laboratory) designation: LrTt1 located on chromosome 2A, LrTt2 located on chromosome 5BL (Leonova et al., 2004, 2010), LrSelG12 located on chromosome 3BL (Singh et al., 2017). Thus, the list of Lr-genes from T. timopheevii is small.

The effectiveness of these genes varies. Thus, the pathogen of leaf rust has virulence to the Lr18 gene in Germany, Switzerland and Russia (McIntosh et al., 1995; Sibikeev et al., 2020), but this gene is more effective in Australia (McIntosh et al., 1995). The MBRL and PNMQ races are virulent to the Lr50 gene at the seedling stage, but this gene is effective at the adult plant stage in Kansas and Texas (Brown-Guedria et al., 2003). The LrTt1 gene is effective at the seedling phase (Leonova et al., 2004), while LrTt2 and LrSelG12 are effective both at seedlings and adult plants (Leonova et al., 2010; Singh et al., 2017).

However, practical wheat breeding has shown little use of these genes in commercial cultivars. Only Lr18 is used in Australia in the Timvera cultivar and its derivatives, and in the Sabikei cultivar and its derivatives (McIntosh et al., 2013). One of the reasons for the insignificant use of introgressions with leaf rust resistance genes from T. timopheevii is their insufficient prebreeding study, which leads to caution in their use by breeders due to fear of linkages with genes that negatively affect agronomic valuable traits.

The purpose of our research was: based on the results of studying the spring T. aestivum/T. timopheevii line L624, to identify its prospects for practical breeding both in terms of effectiveness against P. triticina and in terms of its effect on grain productivity and the quality of flour and bread.

## Materials and methods

The material used included the following genotypes: 1) cultivars of spring bread wheat Saratovskaya 68 (S68), Dobrynya and standard cultivar Favorit; 2) T. aestivum/ T. timopheevii line L624 = Saratovskaya 68/T. timopheevii*4// Dobrynya. The cultivars Saratovskaya 68 and Dobrynya differ from each other by morphotype, presence of different Lr-genes, and flour and dough quality. The first cultivar is awned, red-grained, white-haired, tall, mid-ripening, susceptible to the leaf rust pathogen, contains the ineffective Lr10 gene (Gultyaeva et al., 2020), belongs to the category of valuable wheat in terms of the quality of flour and bread.

The second cultivar is awnless, red-grained, white-haired, tall, mid-ripening, according to the quality of flour and bread, it belongs to the category of strong wheat. The cultivar Dobrynya contains the 7DS-7DL-7Ae#1L translocation from Agropyron elongatum (Host) Beauv. with Lr19/Sr25 genes resistant to leaf and stem rust. These resistance genes have been overcome by pathogens in the Middle and Lower Volga, Central and Central Black Earth regions of Russia (Sibikeev et al., 1996; Baranova et al., 2021).

The standard cultivar Favorit is awnless, red-grained, whitehaired, tall, and mid-ripening, according to the quality of flour and bread, it belongs to the category of valuable wheat. The cultivar is resistant to leaf rust and powdery mildew pathogens and is characterized by the substitution of the bread wheat chromosome 6D by the chromosome 6Agi A. intermedium (Host) Beauv. (Sibikeev et al., 2017).

For production of L624, a sample of T. timopheevii of unknown origin was provided by Dr. S.A. Stepanov (Saratov State University after name N.G. Chernyshevsky, Saratov). T. aestivum/T. timopheevii line L624 was obtained from direct crossing of S68 (female form) with T. timopheevii followed by fourfold backcrossing to the Dobrynya cultivar. Since S68 is susceptible to the leaf rust pathogen, and Dobrynya carries the overcome Lr19 gene, resistance to P. triticina was the main selection criterion during backcrossing. To do this, in artificial infection with P. triticina, we used a population of a pathogen with a high presence of the pp19 pathotype, virulent to Lr19. The stable pathogen-resistant line was isolated from the sixth generation after the last crossing.

The studies included three stages. The first stage was an evaluation of the L624 line resistance to the leaf rust pathogen in the field conditions – the phase of milky-wax ripeness (experimental field of the Agricultural Research Center for Southeast Regions, conditions of strong pathogen epiphytoty in 2017 and of medium pathogen epiphytoty in 2022) and in laboratory conditions – at the seedling phase (third leaf) in 2018–2020. The main differences in P. triticina populations of 2017 and 2022 were that the latter had a decreased percentage of the presence of the pp19 pathotype virulent to the Lr19 gene. In the field conditions, the resistance degree was evaluated according to the scale of A.P. Roelfs et al. (1992), where R is resistance, MR is moderate resistance, MS is moderate susceptibility and S is susceptibility. The degree of rust damage (%) was evaluated according to the scale of R.F. Peterson et al. (1948).

For laboratory evaluation, we used combined Saratov populations of P. triticina with an artificially increased presence of the pp19 pathotype, virulent to the Lr19 gene. Seedlings grown in pots with soil were sprayed with an aqueous suspension of population spores with the addition of Tween 80 detergent. The suspension concentration was 1 mg of inoculum per 1 ml of water. After plant infection, a dark stage was created for 20 hours with 100 % relative humidity, then seedlings were grown at a temperature of 20–22 °C, photoperiod consisted of two stages: day (16 hours) and night (8 hours).

The type of wheat reaction to the pathogen was determined according to the scale of E.B. Mains, H.S. Jackson (1926), where 0 is the absence of symptoms; 0; – necrosis without pustules; 1 – very small pustules surrounded by necrosis; 2 – pustules of medium size, surrounded by necrosis or chlorosis; 3 – pustules of medium size without necrosis; 4 – large pustules without necrosis; X – pustules on the same leaf of different types, chlorosis and necrosis are present. Plants with reaction types 0, 0; 1, 2 were considered resistant (R), and 3, 4 and X (S) were considered susceptible.

The second stage was the cytogenetic evaluation of T. aestivum/T. timopheevii line L624. The purpose of the cytogenetic analysis of the introgressive line was to identify alien genetic material and determine its state in the reconstructed genome of bread wheat, in the form of additional or substituted chromosomes, translocations. This made it possible to evaluate the stability of inheritance of the target trait – resistance to P. triticina.

Preparations of mitotic chromosomes were prepared from the meristem of seedling roots in accordance with the method of E.D. Badaeva et al. (2017). To analyze the L624 line karyotype, we used the FISH method (f luorescent in situ hybridization) using probes based on various repeating sequences. The probe pSc119.2 (Bedbrook et al., 1980) is localized mainly on the chromosomes of the B genome of bread wheat, while pAs1 (Rayburn, Gill, 1986) is localized mainly on the chromosomes of the D genome. Simultaneous use of these probes makes it possible to identify all chromosomes of the B and D genomes, and some chromosomes of the A genome (Schneider et al., 2003). In addition, the chromosomes of the G genome of T. timopheevii can be identified by the localization of hybridization signals with the pSc119.2 probe (Jiang, Gill, 1994).

The repeating sequence Spelt1 was isolated from the genomic DNA of Ae. speltoides Tausch. (Salina et al., 1997); the repeat blocks are localized in the subtelomeric regions of the chromosomes of this species. Individual Spelt1 sites are found in some accessions of tetra- and hexaploid wheats, in particular T. timopheevii, and can serve as markers of these chromosomes (Salina et al., 2006). FISH was performed according to the method of E.A. Salina et al. (2006) with minor modifications. Microscopic analysis was carried out at the Multiple-Access Centre for Microscopy of Biological Subjects (Institute of Cytology and Genetics SB RAS, Novosibirsk, Russia).

In addition to cytogenetic analysis, molecular genetic analysis was performed. Total DNA was isolated from 5–7 day old seedlings by the method of J. Plaschke et al. (1995). Microsatellite markers were used for analysis: Xgwm312 (Röder et al., 1998), most closely located to the LrTt1 resistance gene on the long arm of chromosome 2A (Leonova et al., 2004); markers of the long arm of chromosome 2D – Xgpw4480 (http://www.graingenes.org.) and Xksum73 (Yu et al., 2004). The polymerase chain reaction (PCR) procedure was carried out according to the protocols of the authors. Separation of the amplification products was performed in a 2 % agarose gel.

The third stage was the evaluation of grain productivity traits, physical and baking properties of dough and bread in the recipient cultivars S68, Dobrynya, the standard cultivar Favorit and the T. aestivum/T. timopheevii line L624. The studies were carried out in 2017–2022.

The hydrothermal coefficient (HTC) for the growing season of bread wheat in 2017 was 1.0 (satisfactorily moistened conditions), in 2018 and 2019 the HTC was 0.6 (very dry conditions), in 2020 – 0.8 (dry conditions), in 2021 – 0.9 (dry conditions), and in 2022 – 0.8 (dry conditions). The main differences in weather conditions in 2018, 2019 and 2021 were high temperatures during the flowering period (above the long-term average by 5.0, 4.2 and 8.0 °C, respectively) with a reduced precipitation amount (below the long-term average by 13–15 mm), which sharply reduced grain yield. At moderate temperatures, the most humid was the growing season of 2017, but in 2020, as in 2017, during the flowering phase, the air temperature was lower than the long-term average, namely by 0.7 and 1.0 °C, while the precipitation amount was 23 and 48 mm higher, respectively, in 2017 and 2020, which increased grain yield. The weather conditions in 2022 were characterized by an increased air temperature during the flowering phase by 1.0 °C with a precipitation amount reduced by 12 mm, but in the next ten days (beginning of July) an increase in precipitation by 16 mm was noted at a low temperature by 1.0 °C, which in general allowed to neutralize the harmful effect.

The experimental material was randomly sown in 7 m2 plots in three replicates. The seeding rate was 400 grains per 1 m2. The bread making and flour quality was evaluated by the content of raw gluten, gluten strength and the indicators of the IDK-3 device (deformation index of gluten) and the Chopin alveograph with the baking of experimental bread samples. The protein content of grain, harvested in 2020–2022, was determined on the grain analyzer Infratec TM 1241. The obtained data on T. aestivum/T. timopheevii line L624 and recipient cultivars S68, Dobrynya and the standard cultivar Favorit were subjected to one-way analysis of variance with multiple comparisons according to Duncan using the Agros 2.10 breeding and genetic software package (Martynov et al., 2000).

## Results

Phytopathological analysis of resistance to the leaf rust causative agent

Under strong leaf rust epiphytotic condition in the growing season of 2017, and medium epiphytoty in 2022, line L624 showed a resistant reaction type (R) (severity 0 %, type of reaction – (IT) = 0;), while recipient cultivars: S68 – susceptibility to the pathogen (S, IT = 3+ in 2017 and S, IT = 3 in 2022) (severity 20–40 %) and Dobrynya – susceptibility to the pathogen (S, IT = 3 in 2017 and RS, IT = 0;/3 in 2022). Similar results were obtained during artificial inoculation of cultivars and line L624 at the seedling phase under greenhouse conditions (Table 1).

**Table 1.**
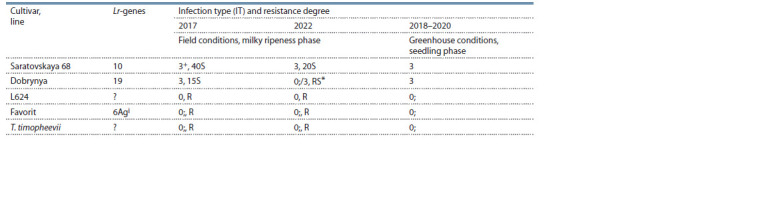
Resistance of line L624, parental cultivars and standard cultivar to P. triticina under field conditions (natural epiphytotics) and under greenhouse conditions (artificial inoculation) This infection type and resistance degree are caused by the low presence of the pp19 pathotype in the P. triticina population.

Thus, the phytopathological analysis of resistance to the leaf rust pathogen T. aestivum/T. timopheevii line L624 under field and laboratory conditions showed its high level in comparison with the recipient parental cultivars and the donor species. It should be clarified that the artificial inoculation of cultivars and L624 was carried out three times during 2018–2020 by Saratov populations of the pathogen with the addition of the pp19 pathotype collected from the infected cultivar Dobrynya. All three evaluations (for 2018, 2019 and 2020) had the same results in terms of ITs for all studied cultivars and L624. The high efficiency of L624 resistance to P. triticina both at the seedlings and at milky ripeness stages indicates the juvenile nature of resistance.

Cytogenetic analysis of T. aestivum/T. timopheevii line L624

**Fig. 1.**
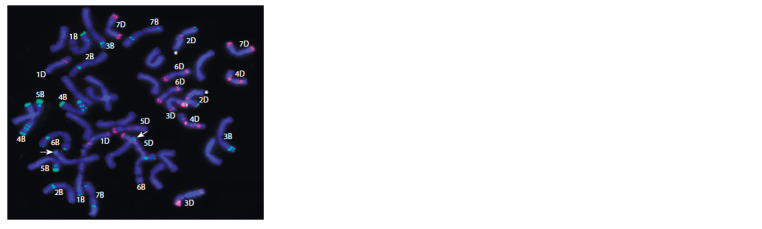
FISH on metaphase chromosomes of the line of spring bread wheat L624 = Saratovskaya 68/T. timopheevii *4//Dobrynya with pSc119.2 probe (green signal) and pAs1 probe (red signal). The arrows indicate the sites of hybridization with the pSc119.2 probe on the long arms of one of the A genome chromosomes. The asterisks indicate the long arms of the 2D chromosomes.

In general, according to the results of cytogenetic analysis, line L624 is cytologically stable and is characterized by a number of chromosomes standard for hexaploid wheat – 42 chromosomes. Chromosomal substitutions and translocations with chromosomes of the T. timopheevii G genome have not been identified. FISH results with pSc119.2 and pAs1 probes on L624 metaphase chromosomes are shown in Fig. 1.

**Fig. 2.**
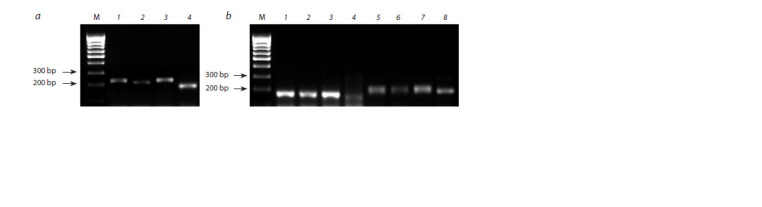
Electrophoregram of the results of PCR amplification of microsatellite markers with DNA of the L624 line and parental forms. a – Xgwm312; b – Xgpw4480 (1–4), Xksum73 (5–8): 1, 5 – T. aestivum (Saratovskaya 68), 2, 6 – T. aestivum (Dobrynya), 3, 7 – line L624, 4, 8 – T. timopheevii. M – marker of fragment length.

Hybridization with pSc119.2 and pAs1 probes did not reveal any changes in the B and D genomes chromosomes of the L624 line, except for chromosome 2D. Chromosome 2D lacks the pAs1 signal at the end of the long arm (Schneider et al., 2003) (see Fig. 1), which may indicate a translocation of unknown origin. The performed molecular genetic analysis showed that when using microsatellite markers of the 2DL chromosome (Xgpw4480, Xksum73) (Fig. 2, b), the L624 line amplification fragments correspond in length to the fragments of control wheat samples. Thus, it can be concluded that the detected translocation from T. timopheevii in the long arm of chromosome 2D does not include the localization area of the Xgpw4480, Xksum73 markers, i. e. it is subtelomeric

**Fig. 3.**
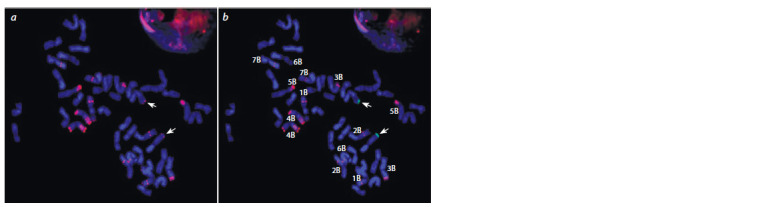
FISH on metaphase chromosomes of spring bread wheat line L624 = Saratovskaya 68/T. timopheevii *4//Dobrynya with pSc119.2 probe (red signal) and Spelt1 probe (green signal). Localization of pSc119.2 probe only is shown on the same metaphase plate (a); arrows indicate pSc119.2 signals on long arms of presumably 2A chromosomes; (b) arrows indicate Spelt1 signals on 2AL.

One of the chromosomes of the A genome had a weak pSc119.2 signal at the end of the long arm (see Fig. 1, Fig. 3, a). Hybridization with pSc119.2 and Spelt1 probes (see Fig. 3) showed that this chromosome also carries the Spelt1 repeat block at the end of the long arm (see Fig. 3, b). Since similar localization of these probes was previously shown on 2Аt chromosomes in some specimens of T. araraticum Jakubz. [syn. T. timopheevii (Zhuk.) Zhuk. subsp. armeniacum (Jakubz.) van Slageren)] (Salina et al., 2006), it could be assumed that the L624 line had the whole 2Аt chromosome of T. timopheevii or a translocation in the long arm of 2A chromosome (T2AS.2AL-2AtL).

However, molecular genetic analysis using the Xgwm312 marker (see Fig. 2, a) showed that the L624 amplification fragment differs in length from the T. timopheevii fragment and corresponds to a fragment of one of the parent wheat cultivars (Saratovskaya 68). This indicates the absence of chromosomal substitution 2At(2A) in line L624 and indicates a terminal translocation in the long arm of chromosome 2A (T2AS.2AL- 2AtL), which does not include the area of localization of the Xgwm312 marker. In addition, there is reason to assume that the Lr gene(s) in L624 are not identical to the LrTt1 gene, which is located close to the Xgwm312 microsatellite marker.

Prebreeding study of T. aestivum/T. timopheevii line L624

The results of the study of grain productivity in T. aestivum/ T. timopheevii line L624, resistant to the leaf rust causative agent, showed that, on average, from 2018 to 2022 there were no significant differences for grain yield in the line compared to the recipient cultivars Saratovskaya 68 and Dobrynya, as well as the standard cultivar Favorit. It is expected, since the productivity traits in 2020 and 2022 are three to five times higher than the yield in 2019 and 2021 (Table 2).

**Table 2.**
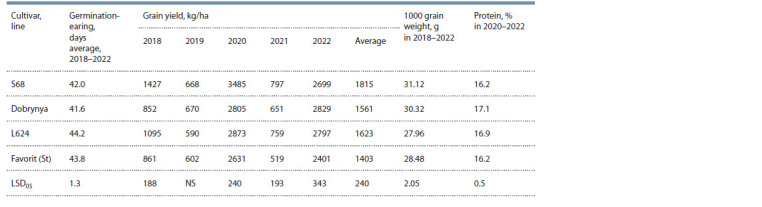
Indicators of grain productivity in T. aestivum/T. timopheevii line L624

However, the analysis of grain productivity by years revealed that out of five years of study, two years of grain yield (2018 and 2020) in L624 were significantly lower than that of the recipient cultivar Saratovskaya 68 and three years (2019, 2021 and 2022) were on the same level. Compared to the cultivar Dobrynya, the grain productivity of L624 was at the same level for four years (2019–2022) and surpassed the recipient cultivar Dobrynya in 2018. Compared to the standard cultivar Favorit, the grain yield of T. aestivum/ T. timopheevii line L624 was higher than of Favorit for four years (2018, 2020–2022) and was at the same level only in 2019 (see Table 2).

As mentioned above, the growing seasons of 2018, 2019 and 2021 were characterized by a hard drought, while the 2020 season was characterized by excess moisture and moderate air temperature from germination to the beginning of flowering, and then a drought with high temperatures was noted until full maturity. At the same time, the 2022 season was characterized by the opposite distribution of precipitation, that is, from germination to the beginning of flowering – a lack of moisture, and during grain filling (July), an excess of moisture. However, the entire period of 2018–2022 relates to arid conditions to varying degrees. Thus, there are grounds to state that L624 has a high drought resistance and that the introgressive material from T. timopheevii has a neutral effect on drought resistance in this line.

On average for 2018–2022, an analysis of 1000 grain weight, as an important element of grain productivity, showed a significant decrease in the line L624 (27.96 g) compared to the cultivars Saratovskaya 68 (31.12 g) and Dobrynya (30.32 g), but a slight decrease compared to the standard cultivar Favorit (28.48 g) (see Table 2).

In terms of the “germination–earing period” trait for the growing seasons of 2018–2022, in L624, compared with recipient cultivars, there were significant differences, namely 44.2 days versus 42.0 days in S68 and 41.6 days in Dobrynya, LSD05 = 1.3 days. At the same time, there were no significant differences with the standard cultivar Favorit (43.8 days).

Plant height of L624 did not differ from S68, Dobrynya, and Favorit. However, lodging resistance differed between L624 (4.24 points) and Dobrynya (4.60 points), but the line did not differ from S68 (4.22 points) and Favorit (4.26 points), LSD05 = 0.15.

The study of the effect of chromosomes or translocations from bread wheat related species on bread making qualities in introgressive lines of bread wheat is an important step in the evaluation of their breeding value. In general, a number of studies of T. aestivum/T. timopheevii lines (Timonova et al., 2012), as well as lines obtained on the spring bread wheat cultivar Saratovskaya 29 using Dr. Savov’s synthetic (AtAtGGDD), revealed a positive or neutral effect of the T. timopheevii genes material on the quality of flour and bread (Laikova et al., 2007, 2013).

In 2020–2022, grain protein content in T. aestivum/ T. timopheevii line L624 was significantly higher than in S68 and Favorit (16.9 versus 16.2 % for cultivars), but was on the same level as Dobrynya (17.1 %) (see Table 2). According to the indicators of the IDK-3 device, gluten content in line L624 was significantly lower than in S68 and Dobrynya, namely 38.7 and 39.7 % versus 36.4 % in L624, with LSD05 = 2.0. At the same time, the line was on the same level with the standard cultivar Favorit (37.2 %). Gluten strength in L624 was significantly higher than in the recipient cultivars and the standard cultivar Favorit, namely 78.1 u.d. against 85.8 (S68), 83.5 (Dobrynya) and 91.3 u.d. (Favorit), LSD05 = 4.0.

When studying the alveograph indicators (Table 3), it was found that dough tenacity, tenacity to extensibility ratio and flour strength in line L624 did not differ significantly from the recipient cultivars. However, all these traits were significantly increased in T. aestivum/T. timopheevii line L624 compared to the standard cultivar Favorit. Crumb porosity and bread volume in L624 were on the same level as in Dobrynya and Favorit, but significantly higher than in S68. Differences in the color of the breadcrumb were observed. Thus, in S68 and L624, the crumb is white, in Favorit, it is cream, and in Dobrynya, it is yellow, which is associated with the presence of 7DS-7DL-7Ae#1L, a translocation that carries genes for high carotenoid content (Prins et al., 1996) (see Table 3).

**Table 3.**
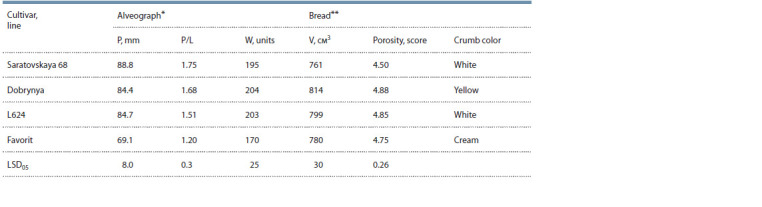
Bread making quality traits in T. aestivum/T. timopheevii line L624 in 2018–2022 * Indicators of the alveograph: P – dough tenacity; P/L – tenacity to extensibility ratio; W – flour strength. ** Indicators of bread evaluation: V – bread volume, porosity.

## Discussion

As noted above, the T. timopheevii species attracts breeders with its high immunity to a complex of fungal diseases. A set of T. aestivum/T. timopheevii lines with resistance genes to various diseases was obtained: to the leaf rust pathogen – Lr18, Lr50, LrTt1, LrTt2, LrSelG12; to the causative agent of stem rust (P. graminis Pers.) – Sr36, Sr37, Sr40; to powdery mildew pathogen (Blumeria graminis DC.) – Pm6, Pm27 (Adonina et al., 2021). The T. aestivum/T. timopheevii lines resistant to pathogens mainly carry introgressive material from chromosomes 6G, 2G, 5G, in addition, from chromosomes 1At, 2At, 3At, 5At, 7At, 3G, 4G, 7G (Badaeva et al., 2010).

In our studies, introgressions from T. timopheevii affected chromosome 2A (T2AS.2AL-2AtL – translocation), and chromosome 2D lacks the pAs1 signal at the end of the long arm, which may indicate a translocation of unknown origin. Previously, in the long arm of chromosome 2A (translocation 2AS-2AtS.2AtL-2AL) of line 842 = Saratovskaya 29 *2/T. timopheevii spp. viticulosum between Xgwm817 and Xgwm312 markers, the LrTt1 gene was mapped (Leonova et al., 2004, 2008, 2011). This gene was found to be inherited in a recessively monogenic manner (Leonova et al., 2004). In L624, the leaf rust resistance gene (according to segregation in the F2 families Saratovskaya 68/T. timopheevii *4//Dobrynya) is also inherited in a recessively monogenic manner (Sibikeev, unpublished data).

However, in our studies, using PCR analysis with the DNA marker of the LrTt1 gene (Xgwm312), it was established that it is not identical to the Lr gene(s) in L624. In addition, LrTt1 is effective against the European population of P. triticina at the seedling stage, but only has a restraining effect (infection type IT = 3) at the stage of adult plants against the West Siberian population of the pathogen (Leonova et al., 2004; Timonova et al., 2012). In addition, translocation with LrTt1 is not linked to resistance to the stem rust causative agent in Western Siberia, the share of damage is 80 % (Timonova et al., 2012).

Introgressions from T. timopheevii in L624 protected against the Saratov population of P. triticina, both at the seedlings stage (three-leaf phase) and at the stage of adult plants (phase of milky ripeness), infection type IT = 0;. Line L624 was affected by the stem rust causative agent at the seedlings stage during artificial inoculation of both the Saratov and Omsk populations, IT = 3.

When trying to identify in L624 genes of resistance to P. graminis (Sr-genes): Sr2, 22, 24, 25, 26, 31, 32, 35, 36, 38, 39, 57 using DNA markers, none of the indicated resistance genes was identified (Baranova, unpublished data). At the same time, earlier in our studies, the effectiveness of L624 (in these studies, L624 is designated by serial number 49) was shown at the seedling stage against the Saratov, Krasnodar, Dagestan and Chelyabinsk populations of P. triticina collected in 2018, as well as against test clones of the pathogen with virulence to the leaf rust resistance genes – Lr9, Lr19, Lr26, the infection type to the pathogen was IT = 0.

In addition, the use of DNA markers for genes Lr1, 3, 9, 10, 19, 20, 21, 22a, 24, 25, 28, 29, 34, 35, 37, 39 = 41, 47, 50, 53, 66, 6Agi made it possible to identify the Lr10 and Lr28 genes in L624. The Lr19 gene from the Dobrynya cultivar was not identified. The detection of the SCS421 gene marker for Lr28 indicates the presence of T. timopheevii (LrTtim) genetic material in the L624 since this marker is not strictly specific for determining the Lr28 gene transferred from Ae. speltoides, and is also present in specimens of T. timopheevii (Gultyaeva et al., 2014). Among the Lr genes from T. timopheevii, the Lr18 gene belongs to the group of ineffective in the conditions of the Volga region and has a different infection type to the pathogen (IT = 3) from IT = 0 in L624.

The infection type of the line with Lr50 (the second Lr gene from T. timopheevii) at inoculation with the Saratov population of the leaf rust pathogen varied from 0–1 to 2+ points and differed from that in L624. The Xgwm382 marker of the Lr50 gene indicated the absence of Lr50 in this line (Gultyaeva et al., 2020). Thus, there are grounds to suggest that L624 in the T2AS.2AL-2AtL translocation contains a leaf rust resistance gene that is different from Lr18, Lr50 and, possibly, non-allelic to the LrTt1 gene.

Analyzing the effect of introgressive material with pathogen resistance genes from T. timopheevii on agronomically important traits, its multidirectional influence should be noted (Leonova, 2018). However, there are very few data on the specific effect of introgressions involving the 2At chromosome on productivity indicators.

There is a study of T. aestivum/T. timopheevii lines 5352- 104 and 5360-191/5, obtained by three backcrosses of hybrid parental lines 744 and 832 (T. aestivum−T. timopheevii, 2n = 42) with Saratovskaya 29 and subsequent self-pollination (Timonova et al., 2012). Line 5352-104 contains introgressive fragments of chromosomes 1At and 2At, while line 5360-191/5 contains 2At and 5GL. In terms of plant height, these lines did not differ from Saratovskaya 29 (S29), and the ear length of the line 5352-104 was higher than that of the S29 cultivar.

According to the number of spikelets per spike, no differences from S29 were found, but in terms of the number and weight of grains per spike, the line’s indicators were higher than those of the recipient cultivar. In terms of grain weight per plant and of 1000 grain weight, line 5352-104 did not differ from S29. Line L 5360-191/5, as a possible carrier of the LrTt1(2At) and LrTt2 (5GL) genes, did not differ from the recipient cultivar S29 in all parameters of ear productivity, as well as grain weight per plant and 1000 grain weight and plant height (Timonova et al., 2012).

In our studies, introgressions in L624 affected chromosomes 2A and 2D, resulting in highly effective leaf rust resistance. Of the five years of studying L624, in 2018 and 2020, the grain yield was significantly lower than that of the recipient cultivar Saratovskaya 68, in 2019, 2021 and 2022, it was on the same level. Compared to Dobrynya, the grain productivity of L624 was at the same level for four years (2019–2022) and surpassed the recipient cultivar Dobrynya in 2018. Compared to the standard cultivar Favorit, the grain yield of T. aestivum/T. timopheevii line L624 surpassed Favorit for four years (2018, 2020–2022) and was on the same level only in 2019. In general, it can be assumed that L624 does not reduce grain productivity. However, by the weight of 1000 grains, L624 was inferior to both recipients and was on the same level with the standard cultivar Favorit. L624 differs from lines L5352-104 and 5360-191/5 in terms of its effect on this trait.

Unfortunately, the results of other researchers on the study of the effect of introgressions involving the 2At chromosome from T. timopheevii on the bread making quality traits is not known to us. It can be stated that introgressions on the 2A and 2D chromosomes in L624 increased the grain protein content compared to the recipient cultivar S68 and the standard cultivar Favorit, but this trait remained at the same level as Dobrynya. In terms of flour and bread quality, L624 was not inferior to cultivar recipients; moreover, it surpassed S68 in terms of bread volume and porosity. At the same time, L624 surpassed the standard cultivar Favorit in all parameters of the alveograph: dough tenacity, tenacity to extensibility ratio and flour strength. Thus, introgressions in chromosomes 2A and 2D in L624 do not impair baking properties. However, the decrease in gluten content in comparison with the recipient cultivar should be noted. For comparison, introgressions from T. timopheevii in the cultivar Pamyati Maystrenko – 2B(2G), 6B(6G) and 1D(1Dt ) increased protein and gluten content, as well as traits of flour strength and bread volume (Laikova et al., 2013).

The 7DS-7DL-7Ae#1L translocation from Ag. elongatum with Lr19/Sr25 genes linked to yellow flour was lost in L624 = Saratovskaya 68/T. timopheevii *4//Dobrynya, despite four backcrosses for the Dobrynya cultivar. As a result, L624 has a white color of flour and bread. The absence of the 7DS-7DL-7Ae#1L translocation in L624 was confirmed using a DNA marker for Lr19/Sr25, SCS265 (Gultyaeva et al., 2020). A possible reason for this is that during the L624 breeding, a constant selection of resistant plants was carried out under the background of inoculation with a population of P. triticina with a high presence of the pp19 pathotype virulent to Lr19.

## Conclusion

In general, regarding the entire complex of agronomic traits, T. aestivum/T. timopheevii line L624 is promising, both in comparison with the recipient cultivars and in comparison with the standard cultivar Favorit. L624 did not reduce grain yield during five years of study, which is apparently due to a fairly high level of drought resistance. The leaf rust resistance gene in L624 is highly effective both at the seedling stage and at the stage of adult plants. For further use of L624 in breeding programs, it is necessary to carry out additional studies on the combination of resistance to P. triticina with resistance genes to the stem rust causative agent, which has become necessary for the Lower Volga region.

## Conflict of interest

The authors declare no conflict of interest.
